# The stability of osseous metastases of the spine in lung cancer – a retrospective analysis of 338 cases

**DOI:** 10.1186/1748-717X-8-200

**Published:** 2013-08-13

**Authors:** Harald Rief, Marc Bischof, Thomas Bruckner, Thomas Welzel, Vasileios Askoxylakis, Stefan Rieken, Katja Lindel, Stephanie Combs, Jürgen Debus

**Affiliations:** 1 Department of Radiation Oncology, German Bone Research Group, University of Heidelberg, Im Neuenheimer Feld 400, 69120 Heidelberg, Germany; 2 Department of Medical Biometry, University Hospital of Heidelberg, Im Neuenheimer Feld 305, 69120 Heidelberg, Germany

**Keywords:** Bone metastases, Spine, Lung cancer, Stability, Fracture

## Abstract

**Background:**

The objective of this retrospective analysis is to systematically assess osseous lesions on the basis of a validated scoring system in terms of stability and fractures prior to and following radiotherapy in 338 lung cancer patients with bone metastases in the vertebral column.

**Methods:**

The stability of 338 patients with 981 osteolytic metastases in the thoracic and lumbar spine was evaluated retrospectively on the basis of the Taneichi-Score between January 2000 and January 2012.

**Results:**

64% (215 patients) were classified stable prior to radiotherapy. Of the stable osseous metastases, none were rated unstable in the further course (p < 0.001, McNemar test). Of the 123 patients in whom the metastases were classified unstable prior to radiotherapy, 21 patients (17%) were classified stable after three months, and 30 patients (24%) stable after six months. A pathological fracture was diagnosed in 62 patients (18%) prior to radiotherapy. Regarding cases of osteolytic metastases of the vertebral bodies in which no fractures could be detected prior to the start of therapy, fractures occurred in 2% of all patients (n = 7) within six months following radiotherapy.

**Conclusions:**

Our analysis demonstrated that pathological fractures following radiotherapy occur in the very minority of vertebral lesions for patients with a favorable outcome. The use of a systematic radiological scoring system to classify osteolytic metastases of the vertebral column has shown to be feasible in daily routine. Prospective clinical trials are warranted in order to analyse, to what extent patients with osseous metastases can be mobilized by physiotherapy for strengthening the paravertebral muscles before radiotherapy effects can be measured by means of radiological recalcification.

## Introduction

Metastases of the bone occur in 30-36% of the patients with lung cancer; 65% of these metastases are discovered at the time of the initial staging [1,2]. The vertebral column is the principal localization of the osseous metastases and is in many cases an indication of an advanced stage of a malignant primary disease [3,4].

Advanced lung cancer is diagnosed in over two thirds of the patients, a figure that correlates with a high mortality [5]. In the majority of patients in lung cancer with bone metastases, the treatment of osteolytic metastases in the daily clinical practice is common. The aim of therapy here is to reduce pain, to improve the functionality, and to prevent complications, for example compression of the spinal cord and pathological fractures. The treatment of osseous metastases is complex and requires a multidisciplinary approach in the form of analgesic therapy, systemic treatment, radiotherapy, and surgical interventions as measures to optimize the prospects of success in the individual therapy of patients [6]. Metastases of the bone play a major role in everyday clinical practice, capable of causing distinct symptoms of pain, bone fractures, compression of the spinal cord, and hypercalcaemia, with a significant reduction in the patients’ quality of life. Metastases occur most frequently as osteolytic factors in connection with bronchial carcinoma and have significant effects on the stability of the vertebral column and the patients’ mobility.

Palliative radiotherapy represents one of the principal therapeutical methods for patients with osseous metastases [4]. The most frequent indications for therapy are the presence of pain, existing or impending instability, neurological symptoms due to compression of the spinal cord, or following surgical intervention [1]. An essential aspect is the stability of the vertebral bodies affected. Patients with unstable osteolytic vertebral bodies are usually given an orthopedic thoracic corset to wear. The pain that is already present is hence additionally compounded by an impairment of mobility and subsequently a further reduction in the patients’ quality of life.

The German Association for Sports Medicine and Prevention and the German Cancer Society have published guidelines for the design of training and sports programs for tumor patients; in these guidelines, the targeted sports intervention is deemed contraindicated in patients with osseous metastases [7]. A systematic classification regarding the stability of the metastases in the vertebral bodies has so far been carried out in only relatively few clinics. The use of a validated scoring system to assess the stability of osseous metastases in the vertebral column may produce early mobilization in palliative-stage patients. The objective of this retrospective analysis is to systematically assess the osseous lesions in terms of stability and fractures prior to and following radiotherapy in bronchial-carcinoma patients with bone metastases.

## Methods

Of a total population of 963 patients whose osseous lesions were treated by radiotherapy at the University Clinic of Heidelberg for osteolytic metastases of the vertebral column due to histologically diagnosed bronchial carcinoma in the period from January 2000 up to January 2012, 338 patients exhibited a survival time exceeding six months; these were examined using computer tomography. This group of patients was screened in this retrospective analysis. Inclusion criteria for the further investigation were radiotherapy performed in the segments afflicted, osteolytic metastases, localization in the thoracic and lumbar spine, and a minimum duration of follow-up treatment of six months. Accordingly 338 patients presenting 981 osseous lesions in the thoracic and lumbar spine were evaluated. Many patients exhibited more than one treated lesion; only one lesion per vertebral body was included in the analysis. The patient data were taken from the Heidelberg NCT Cancer Register. This study was given the positive vote of approval of the Heidelberg Ethics Committee on 22 October 2012.

The diagnosis of osseous metastasis was made on the basis of the findings of computer tomography, magnetic-resonance imaging, or bone-scintigraphy investigations. The patient data are summarized in Table 1.

**Table 1:** Patient characteristics

**Characteristics**		**n**	**%**
Age (years)			
Median (SD)	63,8+/−9,6		
Gender			
Male		232	69
Female		106	31
Karnofsky PS			
40		4	1
50		14	4
60		39	12
70		123	36
80		108	32
90		42	12
100		8	3
Primary site			
NSCLC		303	90
SCLC		35	10
Histology			
Adenocarcinoma		255	75
Squamous cell carcinoma		40	12
Small cell carcinoma		35	10
Large cell carcinoma		8	2
Number of bone metastases			
Solitary		146	43
Multiple		192	57
Spine involvement			
Thoracic		249	74
Lumbar		89	26

*SD* Standard deviation.

The mean age of the patients at the time of the diagnosis of osseous metastasis was 64 years (range 41–88 years). 232 patients (69%) were male and 106 patients (31%) female. Adenocarcinoma was the most frequently diagnosed histological subtype (75%). The performance status was qualified on the basis of the Karnofsky Index [8]. 146 patients (43%) had solitary metastases, while in 192 patients (57%) multiple vertebral bodies were affected.

**Figure 1:**
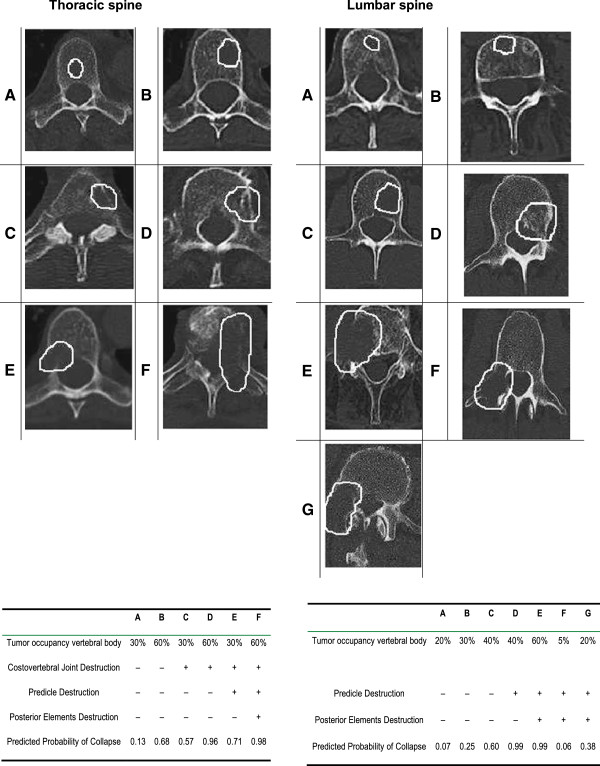
**Taneichi Score.** The stability was assessed according to the Taneichi Score [9].

The stability of each affected vertebral body was assessed according to the Taneichi score [9] on the basis of the CT image recorded before radiotherapy to plan treatment and also in the follow-up stage three and six months after radiotherapy (Figure 1).

The osteolytic metastases were rated on a scale from A to G. Subtypes A to C were defined as stable, subtypes D to G as unstable. In cases in which only one lesion was rated as unstable in a patient with multiple metastases, the score was “unstable”. In 74% of the patients (n = 249; 1–12) the thoracic spine was involved, in 26% (n = 89; 1–12) the lumbar spine. 43% of the patients were equipped with an orthopedic thoracic corset.

In 90% of the cases (303 patients) the primary tumor was an NSCLC, in 10% (35 patients) small-cell bronchial carcinoma. 58% of all patients received chemotherapy prior to their radiotherapy; 85% of the patients were under treatment with bisphosphonates.

### Radiotherapy

The most frequent indication for radiotherapy, in 62% of all cases, was pain; further indications were instability (32%), and neurological symptoms (4%). Radiotherapy post- laminectomy surgery was applied in 2%.

Radiotherapy was carried out at the Department of Radiation Oncology of the University Hospital Heidelberg. After virtual simulation was performed to plan the radiation schedule, radiotherapy was carried out over a dorsal photon field with the energy 6 MV. PTV covered the specific vertebral body affected as well as the ones immediately above and below. 276 patients were treated with 10 × 3 Gy, 24 patients with 14 × 2.5 Gy, 25 patients with 20 × 2 Gy, and 13 patients with 1 × 8 Gy. The median individual dose in all patients was 3 Gy, the median total dose 30 Gy. The respective individual and total doses were calculated separately for each individual patient, depending on the histology, the patient’s general state of health, and the current staging and the respective prognosis (Table 2).

**Table 2:** Treatment characteristics

**Characteristics**		**n**	**%**
Radiotherapy dose completed (Gy)			
Single dose (median, range)		3	(1-4)
Cumulative dose (median, range)		30	(3–40)
Indication for radiotherapy			
Pain		208	62
Instability		108	32
Neurologic		13	4
Postoperative		9	3
Treatment for primary site			
Chemotherapy	Yes	195	58
	No	143	42
Other treatment for bone metastases			
Orthopedic thoracic corset	Yes	145	43
	No	193	57
Bisphosphonates	Yes	288	85
	No	50	15

### Statistical analysis

The empirical distribution of continuous variables is described by the number of observations, mean and standard deviation; the description of categorical variables includes the number and percentage of patients belonging to the relevant categories. Bowker’s test and kappa statistics were calculated to detect possible asymmetry in the distribution of the Taneichi score [9] over time. Graphical methods are applied to present the observed data. Multivariable logistic regression analysis was performed to evaluate possible predictors for stability after six months.

## Results

981 lesions in 338 patients were assessed according to the Taneichi scoring system [9] both prior to radiotherapy as well as three and six months post radiotherapy on the basis of CT images. The median follow up of all patients was 9.3 months (mean 12.2, min. 0.4, max. 130.1 month). The survival status at last follow-up showed 26 patients (8%) still alive and 312 patients (92%) death of disease. The most frequent subtype according to Taneichi was subtype C, with 30%. The mean number of metastases per patient was three [1-12]. 64% (215 patients) were classified stable prior to radiotherapy. Of the stable osseous metastases, none were rated unstable in the further course (p < 0.001, McNemar-test). Of the 123 patients in whom the metastases were classified unstable prior to radiotherapy, 21 patients (17%) were classified stable after three months, and 30 patients (24%) stable after six months. This means that in total 70% and 74% of the osseous metastases were stable after three months and six months, respectively (Table 3). Of the prognostic factors for a therapeutic response already described in the literature on several occasions, although the bisphosphonates did not play a significant role regarding stability (p = 0.074, odds ratio (OR) 1.6), they still appear to exert a positive influence (Table 4). The same model was also used for the aspect of fractures; here, however, no particular parameter showed itself to be a prognostic factor. The evaluation of the distribution of subtypes A to G (Table 5) showed a major change in the direction of improvement over the course of time. Deterioration occurred in 1.8% of the cases (n = 6), improvement in 19.3% (n = 63). No change was seen in 78.9% (n = 258) of the cases. In patients with multiple metastases the most negative subtype was rated, postoperative metastases were not considered (n = 11). This test (Bowker test) shows the distribution pattern of the subtypes according to Taneichi prior to and six months after radiotherapy. Asymmetry was apparent (p = 0.0002) and the correlation (kappa = 0.73) was good.

**Table 3:** The results of Taneichi Score evaluation

**Characteristics**	**n**	**%**
Stability previous RT		
Unstable	123	36
Stable	215	64
Stability after 3 month		
Unstable	102	30
Stable	236	70
Stability after 6 month		
Unstable	93	28
Stable	245	72
Bone fracture before RT		
Yes	62	18
No	276	82
Bone fracture 6 month after RT		
Yes	69	20
No	269	80

**Table 4:** Results of prognostic factors related to stability

**Parameter**	**p-value**	**Odds ratio**	**95% CI**
Age	0.255	0.98	0.959-1.011
Karnofsky-index	0.867	0.99	0.976-1.021
Gender	0.109	1.52	0.909-2.560
Chemotherapy (yes vs. no)	0.692	0.83	0.334-2.074
Primary site (NSCLC vs SCLC)	0.118	0.53	0.246-1.171
Bisphosphonates (yes vs. no)	0.074	1.58	0.957-2.606
Number of metastasis (1 vs. > 1)	0.410	0.79	0.469-1.363

Multivariable regression analysis of prognostic factors related to stability.

**Table 5:** The evaluation of the distribution of subtypes stable and instable metastases over the course of time (0–6 month)

Subtypes before radiotherapy	Subtypes after 6 month								
		A	B	C	D	E	F	G	Total
	A	35	0	0	0	0	0	0	35
	B	11	54	3	0	0	0	0	68
	C	1	12	88	0	0	0	0	101
	D	1	1	5	10	0	0	0	17
	E	4	2	9	2	43	3	0	63
	F	1	1	4	0	6	22	0	34
	G	1	0	1	0	0	1	6	9
	Total	54	70	110	12	49	26	6	327

This Bowker Test showed the distribution of subtypes of Taneichi-Score before and 6 month after radiation therapy. Asymmetry was apparent (p = 0.0002) and the correlation (kappa = 0.73) was good. The evaluation of the distribution of subtypes A to G showed a major change in the direction of improvement over the course of time. Deterioration occurred in 1.8% of the cases (n = 6), improvement in 19.3% (n = 63). No change was seen in 78.9% (n = 258) of the cases.

A pathological fracture was diagnosed in 62 patients (18%) prior to radiotherapy. Regarding cases of osteolytic metastases of the vertebral bodies in which no fracture could be detected prior to the start of therapy, fractures occurred in 2% of the patients (n = 7) within the six months following radiotherapy. Of these 2% (n = 7) six patients were unstable.

## Discussion

An early identification of osseous metastases and their classification in terms of stability is a major factor in the decision for the therapeutical measures to be taken. The scoring system according to Taneichi [9] constitutes a simple method for classifying the vertebral bodies as “stable” or “unstable”, which is why this score is employed in this evaluation.

Osseous metastases in the vertebral bodies are a frequent secondary disease associated with bronchial carcinoma. Palliative percutaneous radiotherapy represents one of the most important measures in this context. Symptoms such as painful mobility impairments, resting pain, fear of pathological fractures, and fatigue result in a substantial reduction in the patients’ quality of life on the one hand; on the other, the treatment of this disease requires protracted therapeutical measures that are highly intensive in terms of cost and time. What is more, some of the patients affected are at an acute risk of fracture, with the potential risk of developing symptoms of paraplegia.

The literature contains various classifications regarding the stability of the vertebral column [9-12]. The essential risk factors for instability comprise an excessive tumor size, the localization of the tumor in the vertebral body, the degree of stress on the vertebral bodies, bone density, the destruction of the costovertebral articulation and of the pedicles in the thoracolumbar spine, and the type of tumor involved [13]. The study by Taneichi et al. [9] defines the risk factors for fractures of the vertebral bodies caused by osteolytic metastases and rates the estimated fractures according to different types of metastatic involvement, establishing criteria for assessing the risk of vertebral-body fractures. The risk factors for vertebral-body fractures in the thoracic region (T1-T10) are the tumor size and the degree of destruction of the costovertebral joint; in the thoracolumbar and lumbar region (T10-L5), it is the tumor size and degree of pedicle destruction that are the main factors.

Our results show that the majority of the patients (64%) already exhibit stable osseous metastases in the vertebral column prior to the start of therapy. Radiotherapy and consecutive resclerotization of the lesions made it possible to reclassify 24% of the originally unstable osteolytic processes as stable after six months. Considering all patients, this means that six months after radiotherapy 72% of the patients can be classified stable. In the literature, the therapeutic response is generally expressed in terms of the quality of pain [14]. The assessment of the therapeutic response using the Taneichi score on the basis of changes in the subtypes has not been described previously. The application of therapeutic measures results in consecutive resclerotization within the osteolytic metastasis, producing an improvement in the Taneichi subtype to the next positive type up on the scale. This change into a better subtype was seen in 19.3% of the lesions. This result represents the therapeutic response of the osteolytic lesions of a bronchial carcinoma following radiotherapy; it is not, however, comparable with the clinical parameters such as pain response.

Bisphosphonates were taken by 85% of the patients, which is why they were not identified as a prognostic factor in terms of stability. Chemotherapy, on the other hand, showed a positive influence in terms of stability and fractures.

The systematic review by Weber et al. [13] defined risk factors regarding spinal instability caused by metastases. Of these risk factors, only the number of metastases showed a positive – albeit insignificant – effect in terms of stability. A uniform classification of spinal metastases in association with bronchial carcinoma has not yet been described in the literature.

In previous retrospective studies among American and Japanese populations, the incidence of pathologic fractures in the vertebral column is given at 10% [1,15]. There are only few data available regarding the risk of fractures of the vertebral bodies following radiotherapy. Our results show a pathological fracture in 18% of the vertebral bodies prior to radiotherapy. Other fractures up to six months in the further course were seen in 2% of all cases. In a further analysis by Saad et al. [16] the risk of pathological fracture in association with lung cancer is given at 17%; this finding, however, was made relative to the entire skeletal system. Pathological fractures are a frequently encountered event; fractures of the vertebral body following radiotherapy, on the other hand are seldom reported.

The vertebral column as the main axis of the human body stands at the center of all the individual’s mobility faculties; when impaired, it constitutes a mobility-restricting factor. Cheville et al. [17] in their study in patients with an advanced tumor disease showed that radiotherapy can be accompanied by physiotherapy, and that the adjunctive effect of this is beneficial. An active mobilization of these patients has so far been contraindicated according to the recommendations of the German Association for Sports Medicine and Prevention and the German Cancer Society [7]. The strengthening of the paravertebral muscles could be a reasonable therapeutical option for patients with stable osseous lesions, and may produce an improvement in their condition in terms of quality of life, fatigue, and pain. Quality of life is an important factor in palliative-stage patients with bronchial carcinoma, and is substantially impaired by pain, fatigue, and loss of mobility. Seen in this light, the classification of stability prior to the start of therapy is of great importance.

Only few clinics have established a uniform systematic classification in terms of the stability of metastases in the vertebral bodies. The high proportion of stable lesions and the negligibly small proportion of fractures make this a potential new option in the palliative treatment of osseous metastases of the vertebral column. The feasibility of a dedicated sports program for these patients is currently the subject of a prospective study by Rief et al. [18]. Our expectation is that the mobilization of this hithertoin adequately considered category of patients will bring about a considerable improvement in the quality of life of patients with osseous metastases who have undergone radiotherapy. One factor that limited the expressiveness of this analysis, however, is the fact that those patients with a survival time of less than six months and other localizations of osseous metastases were not considered. Usually, the benefit from RT regarding bone recalcification is observed after a minimum of 3–6 months, so patients with a shorter survival will not necessarily benefit from this radiotherapeutic effect, and are thus excluded from analysis.

## Conclusions

The use of a systematic radiological scoring system to classify osteolytic metastases of the vertebral column has shown itself to be practicable in the daily routine in the radiology area. The next step that is planned is to use the scoring system for other prospective studies in patients with radiologically treated osseous metastases to validate the physiotherapeutical measures.

## Competing interests

The authors declare that they have no competing interests.

## Authors’ contributions

HR and JD developed and planned the retrospective analysis. TB is responsible for statistical considerations/basis of the analysis. All authors read and approved the final manuscript.

## Authors’ information

Co-authors: Marc Bischof, Thomas Bruckner, Thomas Welzel, Vasileios Askoxylakis, Stefan Rieken, Katja Lindel, Stephanie Combs and Jürgen Debus.
